# Evaluation of a model averaging algorithm for model-informed precision dosing in the context of parameter misspecifications

**DOI:** 10.1007/s10928-026-10064-5

**Published:** 2026-09-22

**Authors:** Sandra Witta, Sebastian G. Wicha

**Affiliations:** Department of Clinical Pharmacy, Institute of Pharmacy, University of Hamburg, Bundesstraße 45, Hamburg, 20146 Germany

**Keywords:** Model-informed precision dosing, Model averaging algorithm, Therapeutic drug monitoring, Population pharmacokinetics, Bayesian forecasting

## Abstract

A primary challenge for integrating model-informed precision dosing (MIPD) in clinical settings is selecting the best-fit model for newly presented patients. A model averaging algorithm (MAA) proposed by Uster et al., addresses this by leveraging multiple models simultaneously. As the ‘library’ of population pharmacokinetic models for a given therapeutic expands, an understanding of how model misspecifications influence MAA can provide insight to guide the optimal selection of model combinations. The present simulation study evaluated the impact of parameter model misspecifications on MAA performance across three scenarios: (1) misspecified structural parameter (θ_CL_), (2) misspecified residual error (σ²), and (3) misspecified interindividual variability on clearance (ω^2^_CL_). Predictive performance for Bayesian forecasting was assessed within each misspecification scenario for single candidate models, a re-estimated model and various combinations of models in MAA. Performance was evaluated for precision and accuracy. The results showed greatest MAA benefit under structural model misspecification, where combining models with opposing bias showed improved predictive performance. In the case of misspecified residual error and interindividual variability (IIV), MAA tended to down-weight models with high residual error or favor those with higher IIV, respectively. Overall, these findings can be used to inform the selection of model combinations to improve MAA performance, supporting more robust MIPD implementation.

**Graphical Abstract:**

**Figure:**
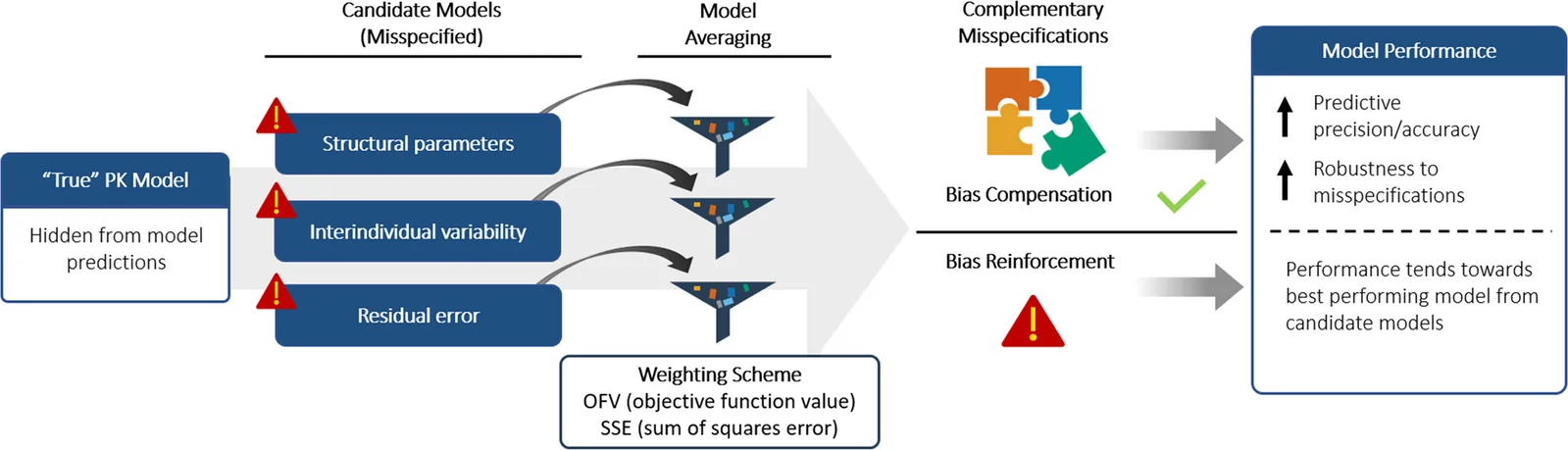

**Supplementary Information:**

The online version contains supplementary material available at https://doi.org/10.1007/s10928-026-10064-5.

## Introduction

Model-informed precision dosing (MIPD) is a rapidly evolving approach for dose individualization through the integration of clinical patient data into mathematical and statistical frameworks [1]. MIPD leverages previously developed population pharmacokinetic (popPK) models describing typical patient pharmacokinetic (PK) profiles, their variability between patients, and parameter-covariate relationships derived from clinical data [2]. MIPD extends beyond conventional therapeutic drug monitoring (TDM) by interpreting TDM data from a patient using these popPK models. Therefore, using the popPK model as prior information and patient covariates together with TDM data, an *a posteriori* estimate of the patient’s PK profile can be derived through a Bayesian approach [2, 3]. Subsequently, the model can be used to calculate a personalized dose to maximize attainment of a predefined target.

The clinical benefits of MIPD, specifically in the context of therapies exhibiting high interpatient PK variability and narrow therapeutic indexes, are highlighted in numerous studies [2, 4, 5]. Particularly, within antimicrobial therapy, linezolid and vancomycin are prime examples of therapeutics with expanding PK model ‘libraries,’ where at least 32 and 63 popPK models exist to date respectively, each founded on unique sets of patient populations [6–9]. Yet, the progression towards MIPD reaches beyond antimicrobial therapy, gaining traction within many therapeutic realms including chemotherapeutic agents [10–12], conditioning therapy prior to stem cell transplantation [13], and immunosuppressants [14].

**Fig. 1:**
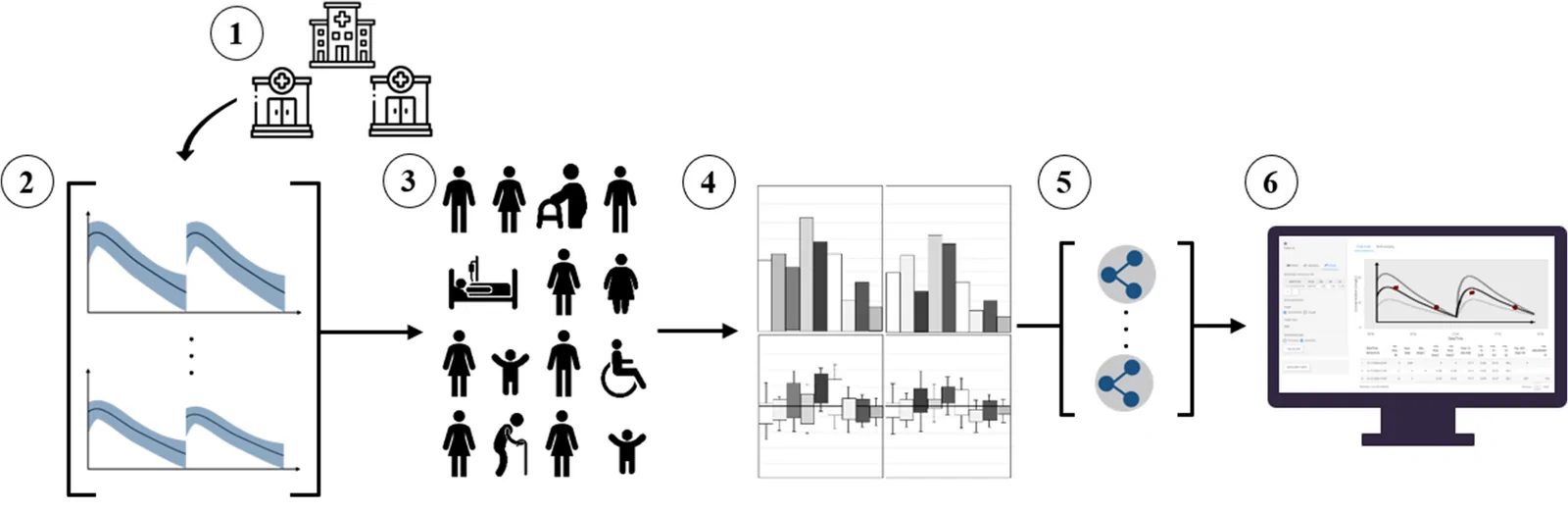
Graphical depiction of the workflow for implementation of reliable MIPD. The process initiates in the clinic (1), where patient data is collected from individuals undergoing therapeutic treatment. Data drawn from distinct patient populations are used to develop individual popPK models (2), resulting in a model library whose parametrization and structure reflects the characteristics of each source population. Candidate models from this library undergo an external model evaluation for the presented patient population (3) to assess model performance with defined metrics (4). An assessment of accuracy and precision is typically used to select a subset of models (5), which can then be integrated into the model averaging approach (6) [18]

Prior to the integration of MIPD in a clinical workflow, an external evaluation of existing popPK models is required to assess their validity on an independent dataset, illustrated in Fig. 1 [15, 16]. Following a thorough assessment based on a variety of predictive performance criteria, the ‘best’ candidate or group of candidate models can be selected. As appropriate model selection presents a challenge to realizing MIPD in a clinical setting, a model averaging algorithm (MAA) previously published by Uster et al. was developed to allow for the simultaneous inclusion of multiple models [17]. The MAA approach assigns weights to each model from a set of candidate models. The weights are thereby reflecting the goodness of fit on the available TDM data until this point in time. Subsequently, a weighted average prediction is calculated across the set of candidate models instead of using only a single model [17]. Weighting schemes are defined by different model fit criteria such as the objective function value (OFV) or the sum of squares error (SSE), reflecting the performance of individual models. This approach provides promising potential towards clinical integration of MIPD as it both alleviates physician or pharmacist burden from meticulous model selection and dampens the risk associated with inappropriate model selection for model-based dose suggestions [17]. However, as the availability of unique popPK models for a given therapeutic continues to broaden, a strategic approach for selecting the optimal number and combination of models within the MAA framework is necessitated to orchestrate a clinically feasible workflow.

As model parameter misspecification can substantially impair predictive performance, understanding how models with different types of parameter misspecification influence MAA may help guide informed selection of model combinations. Therefore, this simulation study aims to evaluate the impact of specific parameter misspecifications on predictive performance, both when models are assessed individually and when incorporated into different MAA model combinations.

## Methods

### Pharmacometric model

**Fig. 2:**
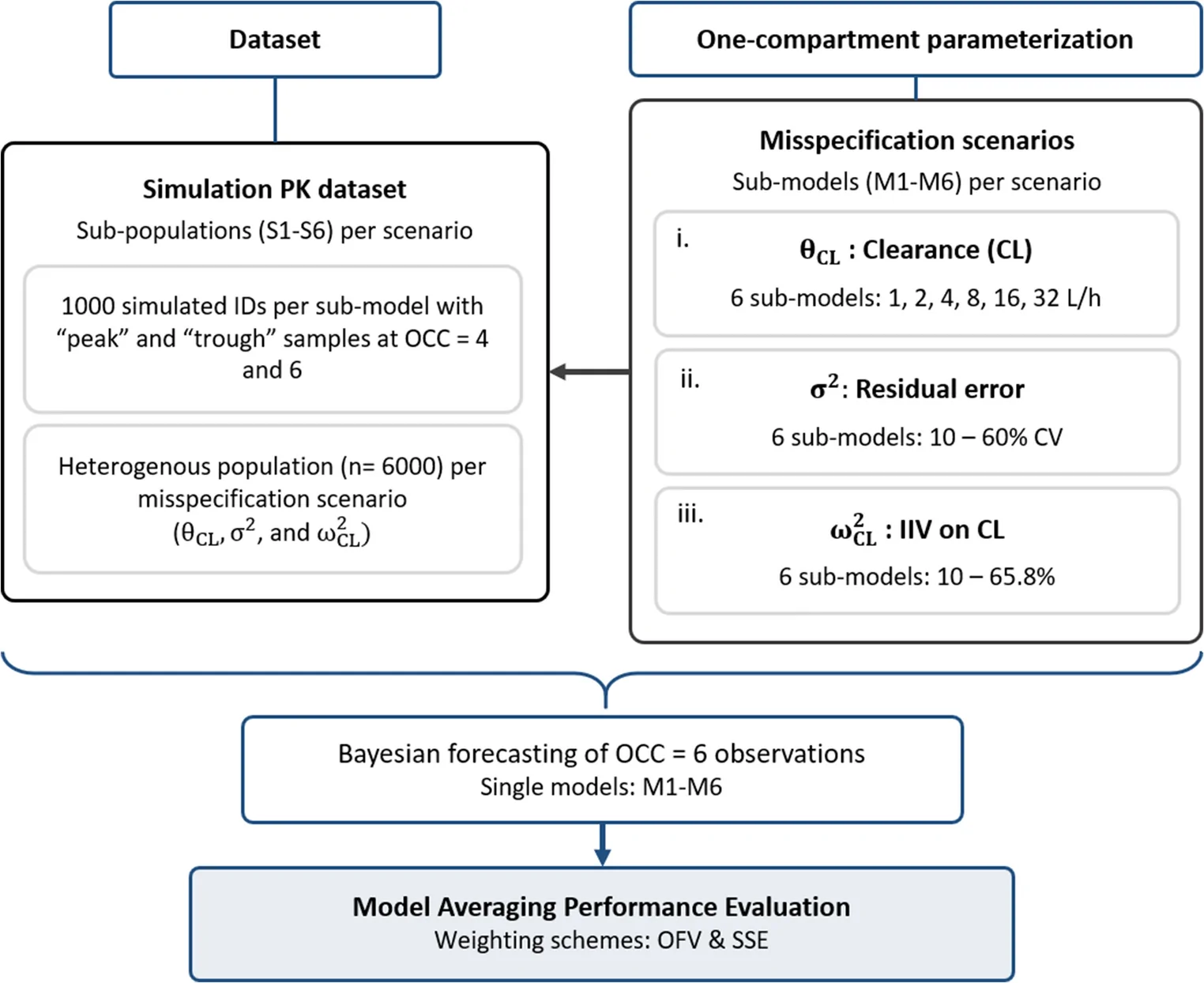
Workflow detailing the steps involved in the generation of heterogenous datasets per simulation scenario. CL is the clearance for a typical individual where values were incremented from 1 to 32 by factor of 2. V_d_ is the volume of distribution of the central compartment for a typical individual; IIV, interindividual variability, is reported as variance and the percent coefficient of variation was calculated as $\:\mathrm{C}\mathrm{V}\mathrm{\%}=\sqrt{{\mathrm{e}\mathrm{x}\mathrm{p}({\upomega\:}}^{2})-1}\mathrm{*}100$. Residual error is expressed as %CV and modeled as proportional error. Model averaging was evaluated using both the objective function value (OFV) and the sum of squares error (SSE) weighting schemes

The workflow of model parameterization and dataset simulation for the presented simulation study is shown in Fig. 2. A one-compartment PK model with linear elimination was parameterized for a hypothetical drug under three types of model misspecification scenarios: (i) variation in the typical clearance value, referred to as structural misspecification (θ), (ii) variation in unexplained residual variability(σ^2^) and (iii.) variation in interindividual variability on clearance (ω^2^_CL_﻿). Within each scenario, six candidate models (M1-M6) were generated by sequentially increasing the parameter of interest from lowest to highest, while keeping all remaining parameters constant at baseline values (θ_CL_: 3 L/h; ﻿θ_Vd_: 60 L; ω^2^_CL_: 0.1; σ^2^: 0.01).

In the θ_CL_ misspecification scenario (i), θ_CL_ was varied according to the sequence θ_CL_ = 2^n-1^, where n is the model number from M1 to M6, yielding θ_CL_ values of 1, 2, 4, 8, 16, and 32 L/h. Clearance values were selected to span a broad range of plausible values in cases when models are selected from diverse population groups to assess robustness of MAA in progressively severe structural misspecification. In the σ^2^ misspecification scenario (ii), residual error variance was increased from (0.1)^2^ to (0.6)^2^, described as a proportional error model corresponding to approximately 10–60 CV%. In ω^2^﻿_CL_ the misspecification scenario (iii), the values for interindividual variability (IIV) on clearance, specified on variance scale, were also incremented from (0.1)^2^ to (0.6)^2^ and assessed by an exponential model corresponding to approximately 10–65.8 CV%. The full model parameterization table is provided in the Supplementary Table S2.

## Simulation of datasets

A dosing schedule and sampling scheme were defined where an initial dose of 2000 mg was administered as an intravenous infusion of 0.5 h, followed by five maintenance doses of 1000 mg every 12 h. Concentrations were simulated according to a sparse sampling design during the 4th and 6th dosing occasions, with samples collected at 1 h post-infusion start (approximate “peak”) and 1 h prior to the next dose (approximate trough). This corresponded to sampling times of 37 and 47 h during the 4th dosing occasion and 61 and 71 h during the 6th dosing occasion. Simulations were performed for 1000 virtual IDs per model, where each of the six models (M1–M6) generated a corresponding sub-population (S1–S6), yielding a dataset of 6000 total IDs per misspecification scenario to mimic a heterogenous dataset. Simulated concentrations with residual variability were regarded as the observed concentrations. Supplementary Table S1 and Fig. S1 provide the dosing template of the simulated dataset structure including dosing information and sampling times, along with visualization of the simulated datasets, respectively.

## Forecasting performance

Predictive performance was assessed using a Bayesian approach where the observed peak and trough concentrations following the 4th dose were considered, yielding *a posteriori* estimates. Concentrations following the 6th dose were blinded to the model and were predicted with Bayesian forecasting and evaluated in comparison to the observed concentrations. Within each scenario (θ_CL_, σ^2^, and ω^2^_CL_) performance was evaluated for: (1) single candidate models (M1–M6), (2) a re-estimated model using first-order conditional estimation with interaction (FOCE-I) on the full (heterogenous) dataset containing simulated data from all six models and (3) various combinations of the six candidate models in MAA. Observed vs. individually predicted concentrations for each of the aforementioned evaluations is provided in Supplementary Fig. S2. Predictive performance was assessed by comparing the predicted and observed concentrations following the 6th dose to quantify the prediction error (PE) of each individual to assess the spread of predictions [15]. The accuracy and precision of the performance were quantified using the median prediction error (MPE) and the median absolute prediction error (MAPE), respectively:

1 $$\:\mathrm{P}{\mathrm{E}}_{\mathrm{i},\mathrm{j}}\left[{\%}\right]=\frac{{\mathrm{C}}_{\mathrm{p}\mathrm{r}\mathrm{e}\mathrm{d},\mathrm{i},\mathrm{j}}-{\mathrm{C}}_{\mathrm{o}\mathrm{b}\mathrm{s},\mathrm{i},\mathrm{j}}}{\left({\mathrm{C}}_{\mathrm{p}\mathrm{r}\mathrm{e}\mathrm{d},\mathrm{i},\mathrm{j}}+{\mathrm{C}}_{\mathrm{o}\mathrm{b}\mathrm{s},\mathrm{i},\mathrm{j}}\right)/2}\:\times\:100$$

2 $$\:\mathrm{M}\mathrm{P}\mathrm{E}\:\left[{\%}\right]=\:\mathrm{m}\mathrm{e}\mathrm{d}\mathrm{i}\mathrm{a}\mathrm{n}\left({\mathrm{P}\mathrm{E}}_{\mathrm{1,1}}\dots\:{\mathrm{P}\mathrm{E}}_{\mathrm{i},\mathrm{j}}\right)\:$$

3 $$\:\mathrm{M}\mathrm{A}\mathrm{P}\mathrm{E}\:\left[{\%}\right]=\:\mathrm{m}\mathrm{e}\mathrm{d}\mathrm{i}\mathrm{a}\mathrm{n}\left(\left|{\mathrm{P}\mathrm{E}}_{\mathrm{1,1}}\right|\dots\:\:\left|{\mathrm{P}\mathrm{E}}_{\mathrm{i},\mathrm{j}}\right|\right)\:$$

pred: predicted; obs: observed; i: individual; j: observation/concentration.

An assessment of relative root mean square error (rRMSE) and relative bias (rBias) is also included in the Supplementary Sect. 1.2.4 (Supplementary Fig. S4) to further evaluate model performance.

## Model averaging and weighting schemes

The model averaging approach is defined on an individual patient level where PK data gathered during TDM is used to first quantify the predictive performance of individual candidate models. Using predefined weighting schemes, the agreement of the fit between the observed and predicted concentrations is assessed to quantify the relative contribution of each model incorporated into the averaging algorithm [17]. The different model weights are then used to calculate the forecast using a weighted average across the candidate models.

The weighting schemes proposed by Uster et al. selected for evaluation within this study were based on the sum of squares error (SSE) and the objective function value (OFV). The SSE weighting scheme relies solely on the deviation between observed and predicted concentrations, while the OFV weighting also considers model structure and provides a more holistic assessment of the goodness-of-fit as it is based on the log-likelihood (LL). Here, the SSE or OFV of the *ith* model is compared relative to the SSE or OFV of the set of *n* models, respectively. In the SSE weighting scheme, obs. represents measured observations while pred. represents the predicted values for the *jth* observation.

4 $$\mathrm{W}_{\mathrm{SSE}_i}=\frac{e^{(-0.5\times \mathrm{SSE}_i)}}{\sum_{1}^{n}e^{(-0.5\times \mathrm{SSE}_n)}}=\frac{e^{\left(-0.5\times\sum\left(\mathrm{C}_{obs,j}-\mathrm{C}_{pred,j}\right)^2\right)}}{\sum_{1}^{n}e^{\left(-0.5\times\sum\left(\mathrm{C}_{obs,j}-\mathrm{C}_{pred,j}\right)^2\right)}}$$

5 $$\mathrm{W}_{\mathrm{OFV}_i}=\frac{\mathrm{LL}_i}{\sum_{1}^{n}\mathrm{LL}_n}=\frac{e^{(-0.5\times \mathrm{OFV}_i)}}{\sum_{1}^{n}e^{(-0.5\times \mathrm{OFV}_n)}}$$

## Combination evaluation

To assess the performance of various model combinations in the MAA approach, six unique combinations were tested according to individual candidate model performance in the θ_CL_ misspecification scenario, in addition to the full combination including all six models. Combinations were systematically selected to assess the impact of model bias as well as the robustness of MAA with respect to the number of models included. Specifically, models were included with only positive bias (M1 and M2), both positive and negative bias (M1 and M6) and the remaining with only negative bias (M3–M6, M4–M6, and M5–M6). The same combinations of models were assessed in the σ^2^ and ω^2^﻿_CL_ misspecification scenarios.

To comprehensively evaluate all possible model combinations in the θ_CL_ misspecification scenario including 2 to 4 models, a total number of 50 unique combinations were assessed.

### Extended simulation analysis

To evaluate the robustness and generalizability of the presented evaluation of the model averaging approach in cases of model misspecification, the simulation study was extended to evaluate both one- and two-compartment models in the context of varying sampling designs. An expanded dataset consisting of dense sampling was simulated for both one- and two-compartment model parameterizations under the previously defined misspecification scenarios (θ_CL_, σ^2^, and ω^2^_CL_). The structural misspecification scenario (for the two-compartment model was extended to additionally include typical value misspecifications for the volume of the central compartment (θ_Vd_) and the intercompartmental clearance (θ_Q_). The one- and two-compartment datasets were subsequently used to assess predictive performance as in the primary analysis under either a sparse or dense sampling design using observed concentrations following either the 1st or 4th dose to predict observations in the 6th dosing occasion. MAA was then applied only in the case of structural misspecification for the sampling design evaluation to assess the generalizability of the results. Finally, a model structure cross evaluation was conducted to assess an additional layer of plausible misspecification. Single model performance was evaluated applying structurally misspecified one-compartment candidate models (θ_CL_) to the dataset generated by structurally misspecified two-compartment candidate models (θ_CL_, θ_Vd_, and θ_Q_) and vice versa. Full details of the simulation design are provided in the Supplementary Material (Sect.  2.1–2.4) with an example of the two-compartment model code provided in Supplementary Code S2.

## Software

Nonlinear mixed-effect modeling with NONMEM^®^ (Version 7.5; Icon Development Solutions, Ellicot City, MD, USA) was used for all dataset simulations, model coding and pharmacokinetic model analysis. Datasets were simulated using the ONLYSIMULATION option with NSUB = 1000. Individual model simulations predicting concentration vs. time data were generated by setting MAXEVAL = 0, while parameter re-estimation was conducted using first-order conditional estimation with interaction (FOCE-I). Post-processing of NONMEM output for model averaging, assessment of predictive performance and graphical visualizations were conducted in R (version 4.5.1). An example of the encoded model structure for the primary study analysis is provided in Supplementary Code S1.

## Results

### Predictive performance

#### **θ**_**CL**_** misspecification scenario**

**Fig. 3:**
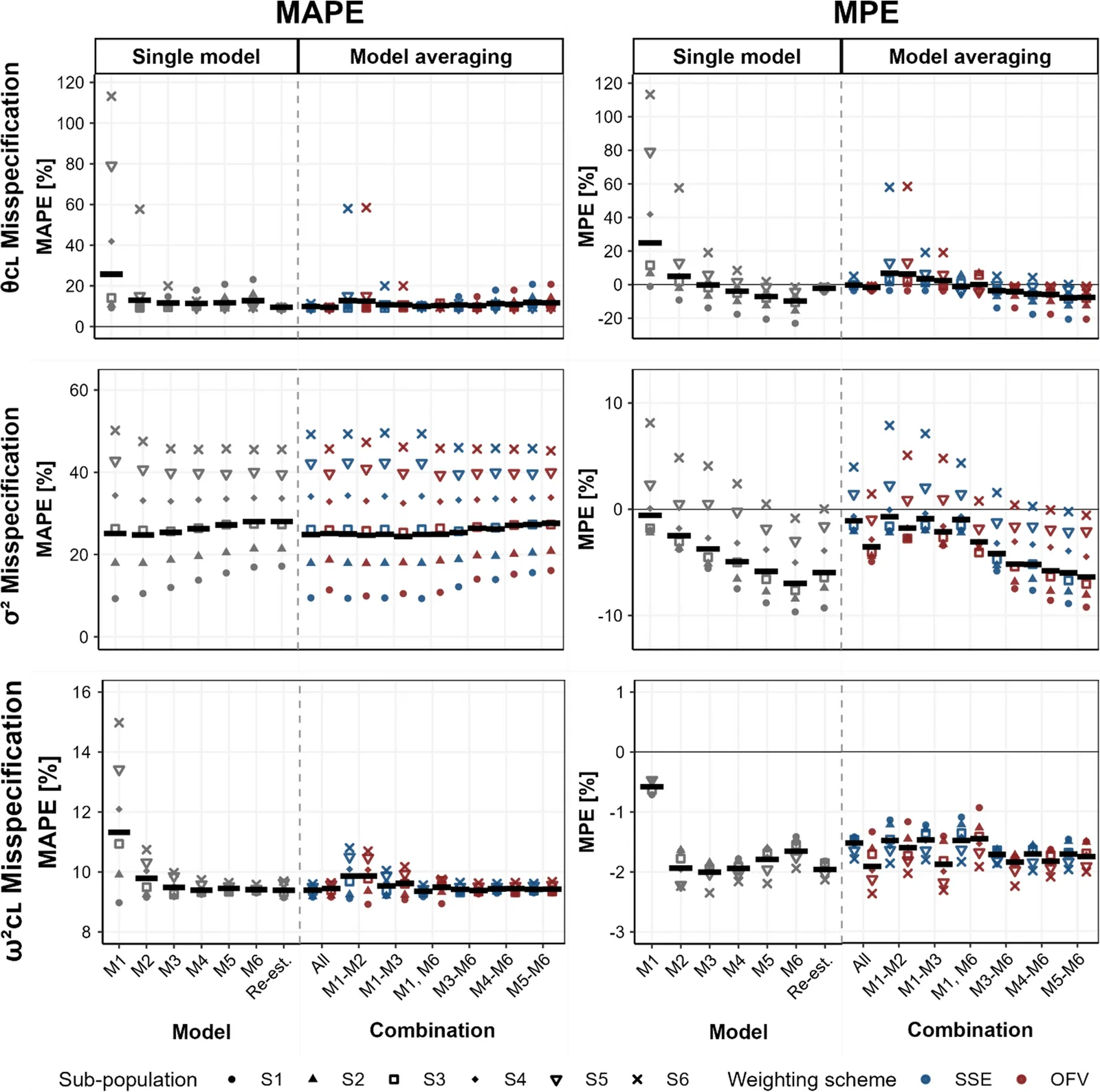
MAPE% and MPE% values from the θ_CL_, σ^2^, and ω^2^_CL_ misspecification scenarios (i.–iii.) based on Bayesian predicted peak and trough concentrations following a 6th dose. Results are shown for (1) single candidate models (M1–M6), (2) a re-estimated model (Re-est.) and (3) model averaging including various combinations of the candidate models, including all six models (M1–M6). Within each misspecification scenario, M1–M6 correspond to parameterizations in which the parameter of interest (θ_CL_, σ^2^, or ω^2^_CL_) was sequentially increased from lowest (M1) to highest (M6), while all other parameters were held constant. In the θ_CL_^2^ misspecification scenario, residual variability (σ^2^) ranges from 0.01–0.36 (0.01, 0.04, 0.09, 0.16, 0.25, 0.36). In the ω^2^﻿_CL_ misspecification scenario, interindividual variability on clearance (ω^2^﻿_CL_) takes the same set of values as in σ^2^. Performance metrics (MAPE/MPE) were calculated on the full population level (*n* = 6000; horizontal line) and on the level of each of the sub-populations, S1–S6 (*n* = 1000; shapes). Within model averaging, blue and red correspond to SSE and OFV weighting, respectively

Overall, the θ_CL_ misspecification scenario (i) shown in Fig. 3 resulted in the largest discrepancies in predictive performance between single candidate models and showed the greatest impact of model averaging performance compared to the σ^2^ and ω^2^﻿_CL_ misspecification scenarios. At the full population level, single-model performance ranged from − 9.74% to 24.8% MPE and 11.4% to 25.7% MAPE. Re-estimating model parameters on the full population improved both accuracy and precision (−2.2% MPE; 9.6% MAPE). At the sub-population level, M1, with the lowest θ_CL_ of all candidate models, showed the greatest variability, with MAPE ranging from 9.20% (sub-population 1) to 113.2% (sub-population 6). Performance improved from M1 to M4, with M4 showing the narrowest range of variability by sub-population (9.45% to 17.9% MAPE) and, at the full population level, the best precision (11.40% MAPE). M3 showed the best accuracy with a MPE of −0.26%. Performance declined for M5 and M6; however, the variability between sub-populations was less pronounced than for M1. For M6, MAPE ranged from 9.61% (sub-population 6) to 23.1% (sub-population 1).

For MAA on the full population level, inclusion of all 6 models using the OFV and SSE weighting schemes (MPE_OFV/SSE_: −1.73%/−0.31%; MAPE_OFV/SSE_: 9.47%/9.81%) approached the performance of the re-estimated model (MPE: −2.21%; MAPE: 9.55%) Pre-selection of different model combinations in the MAA framework, varying both the number of models and the inclusion of models exhibiting opposing bias, revealed considerable differences in performance. Although model re-estimation resulted in the narrowest spread between sub-populations, the inclusion of only 2 of the 6 models with negative and positive bias (M1 and M6) in MAA using OFV and SSE weighting schemes, produced better accuracy than the re-estimated model (MPE_OFV/SSE_: 0.09%/−1.09%).

When combining models of the same polarity in bias, MAA down-weighted the contribution of the less accurate model, approaching the performance of the better performing model. The subsequent inclusion of individually better performing models improved the overall MAA performance. This trend is reflected in the combination of only negatively biased models showing progressive improvement in MPE_OFV/SSE_ (M5–M6: −7.63%/−7.88%, M4–M6: −5.82%/−5.62% and M3–M6: −4.18%/−3.60%, respectively) approaching the accuracy of the best performing individual model.

At the sub-population level, variability in MAA performance was relatively comparable across all combinations evaluated, except for the combination of M1 and M2, where sub-population 6 showed substantially reduced precision and accuracy. For numerical results of all calculated performance metrics see Supplementary Table S3. Additional assessment with calculation or rRMSE and rBias is provided in Supplementary Fig. S4 where similar trends to those for MAPE and MPE were found.

The evaluation of all possible combinations in the case of misspecified clearance (Supplementary Fig. S3) showed varying ranges in accuracy depending on the number of models included. For combinations including two models, the MPE values based on OFV and SSE weighting ranged from −7.63%/−7.88% to 6.27%/6.77% respectively, while for inclusion of 3 models, MPE ranged from −5.82%/−5.62% to 2.38%/3.46% and four models ranged from −4.18%/−3.60% to 0.27%/1.58%. Precision was relatively similar across all combinations. The lowest MAPE value (9.5%) occurred with OFV weighting, resulting from several 4-model combinations and three of the 3-model combinations. The highest MAPE (12.8%) occurred when including two models and applying SSE weighting. Using the OFV weighting scheme, when including two models, the top three model combinations in terms of MPE were M1 and M6 (0.09%), M1 and M5 (0.39%) and M2 and M3 (1.13%). The overall best MPE values were observed for the inclusion of three models with SSE weighting: models M1, M3, M5 (−0.02%) and M2, M3, M4 (−0.04%). Inclusion of four models showed best performance regarding MPE for models: M1, M2, M4, M5 (MPE_SSE_: 0.19%), M1, M3–M5 (MPE_SSE_: 0.21%) and M1–M4 (MPE: 0.27%). Notably, model combinations consistently performed more adequately when M1 was included in the MAA combination. Overall SSE and OFV weighting showed comparable results for each model combination. The spread of prediction error showed comparable interquartile ranges for all combinations of models included in the MAA when using the OFV weighting scheme, with some variability observed using SSE weighting. 

#### **σ**^**2**^** misspecification scenario**

 In the case of σ^2^ misspecification scenario (ii.) shown in Fig. 3, there was comparable precision on the full population level across all single models (MAPE: 24.8% to 28.0%), the re-estimated model (MAPE: 28.1%) and all model averaging combinations regardless of weighting scheme (MAPE_OFV/SSE_: 24.4%/24.9% to 27.6%/27.3%). Accuracy varied for single models, where models with increasing residual error were associated with progressively lower accuracy. This trend was consistent with the IPRED vs. DV diagnostic plots, which demonstrated worsening predictive performance as residual variability increased and a lack of improved performance with model re-estimation (Supplementary Figure S2). Specifically, M1 parameterized with the lowest residual error resulted in an MPE of −0.56%, whereas M6 parameterized with the highest residual error resulted in an MPE of −6.97%. MAA showed best performance with regards to accuracy for combinations including M1 and M2 (MPE_OFV/SSE_: −1.76%/−0.70%), M1–M3 (MPE_OFV/SSE_: −2.11%/−0.88%), and M1 and M6 (MPE_OFV/SSE_: −3.07%/−0.97%), where performance approached that of M1. Notably, all pre-selected combinations for MAA showed greater accuracy than the re-estimated model (MPE: −5.96%) using either weighting scheme when M1 (lowest σ^2^) was included. OFV weighting when all models were included showed slightly less accurate performance compared to SSE weighting (MPE_OFV/SSE_: −3.53%/−1.09%). Overall, combinations including models with lower residual errors showed better performance with SSE weighting having an advantage in predictive performance in comparison to OFV weighting.

On a sub-population level, a comparable trend in precision was observed across all evaluations including single model evaluation, model re-estimation and model averaging, with similar ranges between each sub-population. Performance with respect to accuracy differed slightly more. The least variability was observed in M6 ranging from −9.66% (sub-population 1) to −0.84% MPE (sub-population 6). The largest range occurred in the evaluation of M1 alone, ranging from −1.98% (sub-population 1) to 8.13% MPE (sub-population 6). Within the model averaging approach, the narrowest range in accuracy by sub-population was shown in the case of OFV weighting when M1–M3 were combined (sub-population 1: −3.26%; sub-population 6: 0.78% MPE). See Supplementary Tables S4 for numerical results of all calculated performance metrics. Additional assessment with calculation or rRMSE and rBias is provided in Supplementary Fig. S4. Results indicate that rRMSE is relatively inflated due to sub-populations with small observations that amplify the relative error.

#### **ω**^**2**^_**CL**_** misspecification scenario**

 For ω^2^﻿_CL_ misspecification scenario (iii.), the results in Fig. 3 indicate the smallest overall discrepancies in predictive performance across single model evaluations, model re-estimation and model averaging, compared to the σ^2^ and ω^2^﻿_CL_ misspecification scenarios. The overall scope of calculated precision and accuracy on the full population level ranged from 9.39% to 11.32% MAPE and −2.0% to −0.58% MPE, respectively. The re-estimated model on the full dataset did not display an improved performance compared to the single models (MAPE: 9.39%; MPE: −1.96%). The best performing MAA combination with regards to accuracy was observed for M1 and M6 using OFV weighting (−1.46%), performing slightly better than when all models were included (MPE_OFV/SSE_: −1.92%/−1.53%). Overall however, OFV and SSE weighting resulted in comparable performance. See Supplementary Table S5 for all numerical results.

#### Weighting schemes

**Fig. 4:**
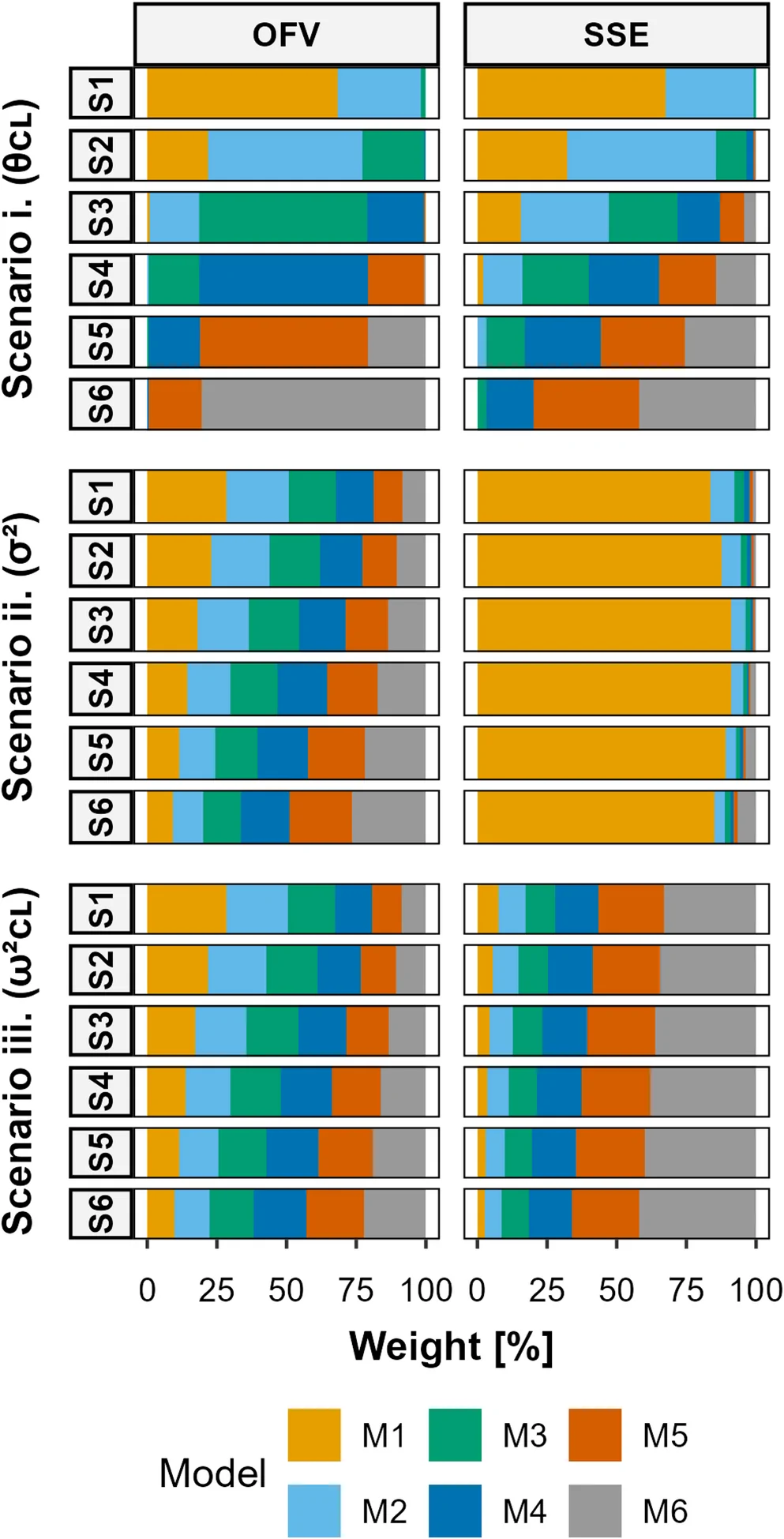
Contribution of sub-models (M1–M6) in the model averaging algorithm approach for all three model misspecification scenarios (i.–iii): structural parameter (θ_CL_), residual variability (σ^2^), and inter-individual variability (ω^2^﻿_CL_). Scenarios are stratified by respective simulated sub-populations (S1–S6) for each model (y-axis). Weight percentages are shown for both OFV and SSE weighting schemes used in MAA

In Fig. 4, the evaluation of the contribution of models in the model averaging algorithm approach showed that the OFV weighting scheme tended to consistently apply a higher weight to the correct model used to generate the respective dataset, across all three scenarios (i.–iii.). This was most notably shown for the θ_CL_ misspecification scenario (i.), where the majority of the weight for each sub-population was assigned to the “true” underlying model. A similar trend was apparent for SSE weighting in scenario i., however, less pronounced as the weights were more distributed across multiple models. This was especially apparent for sub-population 3 to sub-population 6, where a majority of the weights were not concentrated on a single dominant model, in contrast to the OFV weighting scheme. In the σ^2^ misspecification scenario, for all stratified scenarios according to the respective datasets, M1 heavily dominated the overall weighting given an SSE weighting scheme, indicating a strong preference for models with respectively smaller residual errors. Within the ω^2^﻿_CL_ misspecification scenario, the SSE weighting scheme also deviated from the trend observed in θ_CL_ misspecification, where a stronger preference was applied to M6 which was parameterized with a larger inter-individual variability.

### Extended simulation analysis

The single model predictive performance for the one-compartment model showed comparable trends across all evaluated sampling designs, as shown in Supplementary Fig. S9. Sparse sampling using the 1st dosing occasion to predict the 6th dosing occasion showed consistently the least adequate performance across almost all misspecification scenarios, excluding σ^2^ misspecification, where the MPE values for all individually evaluated models and the re-estimated model showed improved predictive performance. In contrast, dense sampling from the 4th dosing occasion consistently showed best performance with regards to MAPE% and MPE%. Supplementary Fig. S10 reports the one-compartment model averaging performance for all previously evaluated combinations with respect to each of the four sampling designs given misspecification in θ_CL_. The results indicate that dense sampling design using the 4th occasion observations performed the best across all model combinations evaluated. The combination of models M1 and M6, with the greatest opposing bias when evaluated individually, improved model performance for all sampling designs with notably some discrepancy in the sparse sampling using the 1st occasion samples, resulting in opposing biases using OFV and SSE weighting schemes.

The single model predictive performance for a two-compartment model structure showed more notable discrepancies between single model performance in the θ misspecification case (Supplementary Fig. S11) compared to either σ^2^ or ω^2^﻿_CL_ misspecification (Supplementary Fig. S12), as in the primary one-compartment evaluation. The θ misspecification case showed a comparable trend to the one-compartment model (Supplementary Fig. S9) for models M1–M6 with subsequently increasing CL values. Misspecification in either the volume of the central compartment (θ_Vd_ or intercompartmental clearance (θ_Q_) showed minimal impact overall (M7–M10). However, misspecification in the intercompartmental clearance (M10) was most prominently identified under the dense sampling scheme using the 1st occasion observations, with negligible impact under the sparse sampling schemes. Model averaging performance using different combinations of varying structurally misspecified models is shown in Supplementary Fig. S13. Notably, when using 1st occasion observations (OCC = 1) in either the sparse or dense sampling scheme, the predictive performance was worse using the OFV weighting scheme than SSE when combining M1 with either M6 or M7, resulting in a lack of bias compensation. Using observations from the more recent occasion (OCC = 4) provided substantially improved predictions. Furthermore, when structural misspecifications occurred on different parameters such as θ_CL_ and θ_Vd_, where the value for θ_CL_ was considerably low (M1) and the volume of the central compartment was also relatively low (M7), there was an observed discrepancy in the scale of opposing bias, with M7 showing only slight negative bias. However, MAA enabled the weighting to allow for the predictive performance of the M1 and M7 combination to gravitate towards that of the higher performing model (M7). The best performing combination regardless of sampling design was the combination of M1, M3 and M10 ( θ_CL,M1_ = 1 L/h, θ_CL,M3_ = 4 L/h, θ_Q,M10_ = 20 L/h, respectively).

Lastly, the model structure cross evaluation, where model performance was evaluated applying structurally misspecified one-compartment candidate models (θ_CL_) to the dataset generated by structurally misspecified two-compartment candidate models (θ_CL_, θ_Vd_ and θ_Q_) and vice versa, showed comparable trends as in the “original design.” However, the MAPE for models with two-compartment model structure evaluated on one-compartment generated data indicated consistently worse performance in the cross-evaluation, most substantially for M1–M7.

## Discussion

A primary barrier to bridging population pharmacokinetic model development and integration into MIPD clinical workflows is the selection of an appropriate model when developing a population specific model is not feasible due to limited expertise, data, or resources. External model evaluations have therefore become an essential step in identifying the most suitable model for extrapolating to new patients at external clinical institutions. Model averaging offers a means to support this process by incorporating multiple models, thereby reducing the burden of single model selection and the associated potential bias. Because model misspecifications are a key source of variability in predictive performance, the present simulation study aimed to systematically investigate the impact of misspecified model parameterizations and provide insight into strategies and considerations for combining individual models in the MAA approach.

This study showed that inclusion of only two models in MAA with strong opposing bias due to structural misspecification on a single parameter resulted in accuracy and precision comparable to the inclusion of all six models and that of a re-estimated model. Of note, in clinical practice, a re-estimated local model is often not available while MAA uses existing literature models. When including model combinations with models exhibiting unidirectional bias, the overall MAA performance approached that of the best performing model. In the case of data with heterogenous residual error, the re-estimated model showed slightly less adequate performance than the MAA approach where models with lower residual errors were included. Although performance differences between OFV and SSE weighting schemes were minor in most cases, SSE weighting showed considerable preference for models with low residual error. This is consistent with the study by Berrah et al., which found that a lower residual error in general can improve the forecasting performance of pharmacometric models applied in the MIPD setting [19]. Lastly, in the case of misspecifications in the parameterization of IIV on a PK parameter, the impact of MAA was minimal; however, there were prominent differences in model contributions between the OFV and SSE weighting schemes, where SSE weighting preferred models with greater values of IIV.

A re-estimated model was used as a benchmark for “ideal” model performance to compare predictive performance between single and model averaging combinations in different cases of misspecification. Although this was selected to reflect a clinically relevant context where a step-wise workflow of developing a new model is not feasible, the re-estimated model performance likely does not attain the predictive performance of an ideally population-specific model. A similar approach is reported in prior studies such as in Bartelink et al., where a previously developed busulfan popPK model is applied to a more diverse, external pediatric population where the model is refit resulting in new parameter estimates of the same model structure [20]. Further studies have developed more elaborate strategies for re-estimating model parameters on real world routine clinical care data, including Hughes et al., 2020 and Maier et al., 2021, where frameworks for continuous learning approaches provide increasing potential for implementation of MIPD [2, 21].

The advantage of model averaging performance in externally evaluated models has been demonstrated in previous studies. In an external model evaluation of 18 infliximab popPK models, Kantasiripitak et al. showed that the inclusion of only four candidate models in a multi-modal evaluation, three with negative rBias and the best model with positive rBias, yielded a predictive performance comparable to that achieved with including all evaluated models in MAA [22]. Similarly, Schatz et al. applied MAA to the external model evaluation of 24 piperacillin popPK models conducted by Greppmair et al., assessing three distinct pre-selection scenarios: (1) inclusion of all models, (2) inclusion of only the best performing models, and (3) inclusion of five models with differing degrees and polarities of bias, including one unbiased model [15, 23]. The predictive performance was found to be best when one sample was included in Bayesian forecasting and when all models were included in MAA, compared to the other two pre-selection scenarios (MPE_All/best/diff_ Bayesian 1: 0.39%/−1.60%/3.19%) [23].

The current study builds on these findings by systematically evaluating the impact of underlying model misspecifications on MAA performance when different combinations of models are selected. In the reduced model sets used for MAA by Schatz et al. and Kantasiripitak et al., which included five and four models, respectively, the pre-selected reduced combinations integrated into MAA included the best-performing, minimally biased model [22, 23]. While these studies may suggest model performance is largely driven by the inclusion of better performing models, findings by Uster et al. indicate that MAA robustness can persist even when only three of the worst performing (two with negative rBias and one positive rBias) candidate models are included, provided that they exhibit opposing bias directions [17]. The results of the present study thus highlight the role of bias compensation when misspecified models with opposing bias are integrated into MAA.

In this simulation study, all possible combinations of candidate models were evaluated under the case of θ_CL_ misspecification which showed the largest discrepancies of single model performance in comparison to the σ^2^ and ω^2^﻿_CL_ misspecification scenarios. Notably, the inclusion of only less adequately performing models resulted in superior performance to the inclusion of all models or the re-estimated model when the models had strongly opposing polarity. This finding can particularly be applied to cases where an external model evaluation results in a lack of adequately performing models, with a mix of models having tendencies to under- and overpredict. For example, in the external model evaluation by Yang et al., all 14 meropenem popPK models failed to meet predefined performance criteria when externally evaluated in a cohort of critically ill Chinese patients, with an rBias for a priori predictions ranging from −302.96% to 130.37% [24]. The external model evaluation conducted by Guo et al. of popPK models for vancomycin in ICU patients from two separate clinical institutions, was limited to only one of the six models reliably predicting the patient PK [25].

From the presented findings, MAA can provide a framework to leverage even relatively poor performing models, which can particularly be applied in cases where no single model adequately describes the population. As described, the inclusion of opposingly biased models may yield more reliable predictions by partially “averaging out” a systematic error. In contrast, when all candidate models show unidirectional bias, MAA will likely be unable to correct the underlying misspecification. For example, in an external model evaluation of posaconazole popPK models, Huang et al. stratified the predictive performance of candidate models by inclusion of proton pump inhibitors (PPIs) as a covariate effect. Models including PPIs showed improved performance, but consistently negative bias, indicating a systematic underprediction [26]. In such a case, including models sharing a negative bias, the overall performance will tend towards the best performing model. This behavior was evident in the θ_CL_ misspecification scenario where varying the number of negatively biased models included in MAA (M3−M6, M4−M6 and M5−M6) resulted in a predictive performance tending towards that of the best performing model within the ensemble. Although, the remaining models in Huang et al. without PPI inclusion showed overall poorer performance, their opposing tendency to overpredict could still be leveraged and potentially help alleviate the model misspecification through bias compensation [26].

The extended simulation analysis assessed whether the findings observed in the primary evaluation, based on model misspecifications given a one-compartmental structure, generalize to a two-compartment structure in the context of varying sampling scenarios. Combining models with opposing misspecification on a single parameter (θ_CL_: M1 and M6) reproduced the bias compensation given a two-compartment model structure, although it was sensitive to the sampling design (Supplementary Fig. S13). In contrast to using observations from a recent dosing occasion (OCC = 4), using observations from the 1st occasion (OCC = 1) resulted in minimal compensation in bias and divergence in predictive performance between the two weighting schemes. In contrast to θ_CL_ misspecification, misspecifications in either the volume of distribution (θ_Vd_: M7 and M8) or intercompartmental clearance (θ_Q_: M9 and M10) produced a smaller range of predictive bias (Supplementary Fig. S11). When combining M1 with M7, the latter characterized by an under-estimated θ_Vd_, MAA was unable to successfully “cancel” out the opposing bias due to a discrepancy in scale of the misspecifications, and thus the overall predictive performance was dominated by the strong positive bias of M1. Therefore, informing the pre-selection of models in MAA with the findings from this study, a strategic approach for identifying when MAA is most optimally used for combining opposingly biased models can potentially provide more robust predictions.

Regarding the θ_CL_ misspecification, the clearance range of 1–32 L/h was intentionally chosen to capture a plausible range of clearance values across diverse patient groups and reflect a heterogenous population. Beta-lactams for example, such as meropenem and piperacillin, are particularly characterized by broad variabilities of clearance in special populations [27–29]. Burn victims particularly, demonstrate augmented renal clearance and dramatic alterations in piperacillin PK resulting in greater risk of subtherapeutic exposure [27, 30]. Contrastingly, patients with end-stage kidney disease undergoing hemodialysis show a reduction of 63% and 79% piperacillin/tazobactam endogenous clearance with and without residual disease in comparison to patients with normal kidney function [31]. PopPK models developed on such special populations are thus prone to having varying systematic bias when evaluated in an external population. Integrating the MAA approach provides greater flexibility, dampening the risk of selecting an inappropriate model unfit to capture diverse patients.

Overall, there were minor discrepancies between the OFV and SSE weighting schemes, with the most prominent difference being in the σ^2^ misspecification scenario. With regards to the accuracy, SSE weighting performed better for all combinations evaluated in the case of σ^2^misspecification. This is in contrast with two previous studies where OFV was found to have overall greater advantages, yet in agreement with the work conducted by Kantasiripitak et al. for the implementation of a multi-model MIPD tool for infliximab which found SSE weighting to be superior [17, 23, 32]. When assessing weight contributions for the two weighting schemes, SSE weighting showed a prominent preference for M1 (lowest σ^2^) in the case of σ^2^ misspecification. This tendency to allocate greater weight to models with lower residual error is consistent with SSE weighting which penalizes models with greater deviations between observed and predicted values. SSE weighting in the case of the ω^2^﻿_CL_misspecification scenarios also deviated from the ability to “find the correct model” where a greater weight was consistently allocated to models with greater interindividual variability on clearance. The tendency for SSE weighting to prefer models with an inflated interindividual variability on clearance is supported by the concept of “flattened priors” [33]. This behavior is attributed to the increased flexibility to capture individuals deviating further from the typical population PK, thus resulting in lower deviations between the observed and predicted values. The application of the concept of “flattened prior” has been previously assessed in Hughes et al. discussing the appropriate context of use and potential risks with overfitting [33]. In summary, the results suggest that preference between OFV and SSE weighting is influenced by the type of anticipated misspecification and influence of sampling design. In the case of structural misspecification, the, OFV and SSE perform overall comparably; however, in the two-compartment θ misspecification scenario under a sampling design using observations from an earlier occasion (1st occasion), OFV and SSE showed divergence when combining models with the most opposing bias (combination: M1, M6; Supplementary Fig. S13). A preference for SSE weighting was evident in the case of heterogenous residual error; however, due to the aforementioned discrepancies in previous studies, further work detailing selection of weighting schemes is necessitated.

The study has several limitations to disclose. First, the results rely solely on simulated data where a single parameter, corresponding to the misspecification scenario, was incremented to generate a heterogenous patient population. In reality however, heterogeneity in populations arises from simultaneous variations in multiple parameters Second, MAA relies on individual concentration data to update the OFV- and SSE-based weighting schemes used to derive model weights. In an a priori scenario, there is a lack of individual-specific information and all models are therefore equally weighted across the model set, unless more sophisticated approaches to calculate the weights are used [34]. Yet, this was outside of the scope of our study which solely evaluated a Bayesian forecasting scenario; however, Uster et al. previously demonstrated the progressive updating of weights with the incorporation of observations from additional dosing occasions ultimately converging towards the underlying “true” simulation model [17]. Third, prospective work to assess the effect of the inclusion and omission of covariates, forms of covariate implementation, or misspecifications in error models would provide a more comprehensive evaluation to better capture the complexity present in clinical populations. Fourth, although the total of 50 possible model combinations given 6 candidate models was assessed within a reasonable time frame, as libraries for a given therapeutic expand, the time to compute needs to be considered. Benchmarking of computation time showed that the inclusion of two models in MAA required an average of approximately 5 min 51 s for a population size of 6000, while the inclusion of four models resulted in an average runtime of approximately 13 min 12 s. Although these times are unlikely to hinder clinical decision making, they present a scalability consideration as runtimes expand, highlighting importance of informed selection of model combinations. Lastly, the assessment of model performance was also limited to quantifying the accuracy and precision of the predictions using MPE and MAPE metrics, respectively. To reliably implement candidate models into a clinical workflow, further assessment is necessitated such as quantifying theoretical target attainments to evaluate the potential clinical impact [9].

## Conclusions

In conclusion, the presented simulation study provides insight into the effect of model parameter misspecifications in the context of MAA. The key findings can be applied in devising an improved strategy for the pre-selection of models to combine for an improved and more robust prediction in the case when the capacity for developing a new population specific model is not available. The work herein thus aims to facilitate the optimal use of the MAA approach when previously published popPK models are integrated into an MIPD clinical workflow.

## Supplementary Information

Below is the link to the electronic supplementary material.

Supplementary Material 1

## Data Availability

No datasets were generated or analysed during the current study.
