# Supplementary material for: Evaluation of a model averaging algorithm for model-informed precision dosing in the context of parameter misspecifications
Source: J Pharmacokinet Pharmacodyn. 2026 Sep 22;53(6):54. doi: 10.1007/s10928-026-10064-5 (PMC13597597; doi:10.1007/s10928-026-10064-5)
Supplement: Supplementary file 1 [file 10928_2026_10064_MOESM1_ESM.docx]

**Title:**

**Evaluation of a model averaging algorithm for model-informed precision dosing in the context of parameter misspecifications**

**Journal Name:**

Journal of Pharmacokinetics and Pharmacodynamics

**Authors:**

Sandra Witta^1^, Sebastian G. Wicha^1^

^1^Department of Clinical Pharmacy, Institute of Pharmacy, University of Hamburg, Hamburg, Germany

**Corresponding author:**

Prof. Dr. Sebastian G. Wicha

Professor of Clinical Pharmacy

University of Hamburg

Institute of Pharmacy

Bundesstraße 45

20146 Hamburg, Germany

sebastian.wicha@uni-hamburg.de

Phone: +49 40 42838 3487

Contents

[1.	One-compartment model simulations	2](#_Toc234963819)

[1.1	Dataset Simulation	2](#_Toc234963820)

[1.2	One-compartment model parameterization and dataset visualization	3](#_Toc234963821)

[1.2	Results	4](#_Toc234963822)

[1.2.1	Goodness-of-fit plots	4](#_Toc234963823)

[1.2.2	MAPE and MPE: raw results	5](#_Toc234963824)

[1.2.3	MAPE, MPE and PE: all possible combinations	11](#_Toc234963825)

[1.2.4	rRMSE and rBias	12](#_Toc234963826)

[2.	Extended simulation analysis: One- and Two-compartment Sensitivity Analysis	14](#_Toc234963827)

[2.1	Overview	14](#_Toc234963828)

[2.2	Dataset simulation	15](#_Toc234963829)

[2.3	Sampling Design	16](#_Toc234963830)

[2.4	One- and two compartment model parameterizations	18](#_Toc234963831)

[2.5	Results	19](#_Toc234963832)

[2.5.1	Impact of sampling design	19](#_Toc234963833)

[2.5.1.1	One-compartment sampling evaluation: single model predictive performance	19](#_Toc234963834)

[2.5.1.2	One-compartment sampling evaluation: model averaging	20](#_Toc234963835)

[2.5.1.3	Two-compartment sampling evaluation: single model predictive performance	21](#_Toc234963836)

[2.5.1.4	Two-compartment sampling evaluation: model averaging	23](#_Toc234963837)

[2.5.2	Model structure cross evaluation: $\theta$ misspecification	24](#_Toc234963838)

# 1. One-compartment model simulations

## 1.1 Dataset Simulation

**Supplementary Table S1.** Dosing template used to simulate datasets for all three misspecification scenarios. Sampling at “peak” and “trough” concentrations were simulated after the 4^th^ and 6^th^ dosing occasions. Following simulation of the datasets, the EVID and MDV values associated with the “peak” and “trough” concentrations following the 6^th^ dose were updated to values of 2 and 1, respectively, to hide the observations during Bayesian forecasting.

| **ID** | **TIME** | **CMT** | **EVID** | **AMT** | **RATE** | **DV** | **OCC** | **GROUP** | **MDV** |
| --- | --- | --- | --- | --- | --- | --- | --- | --- | --- |
| 1 | 0 | 1 | 1 | 2000 | 4000 | . | 1 | 1 | 1 |
| 1 | 12 | 1 | 1 | 1000 | 2000 | . | 2 | 1 | 1 |
| 1 | 24 | 1 | 1 | 1000 | 2000 | . | 3 | 1 | 1 |
| 1 | 36 | 1 | 1 | 1000 | 2000 | . | 4 | 1 | 1 |
| 1 | 37 | 1 | 0 | 0 | 0 | . | 4 | 1 | 0 |
| 1 | 47 | 1 | 0 | 0 | 0 | . | 4 | 1 | 0 |
| 1 | 48 | 1 | 1 | 1000 | 2000 | . | 5 | 1 | 1 |
| 1 | 60 | 1 | 1 | 1000 | 2000 | . | 6 | 1 | 1 |
| 1 | 61 | 1 | 0 | 0 | 0 | . | 6 | 1 | 0 |
| 1 | 71 | 1 | 0 | 0 | 0 | . | 6 | 1 | 0 |

## 1.2 **One-compartment model parameterization and dataset visualization**

Supplementary Table S2. Model parameterization of the one-compartment model for all three misspecification scenarios ($\theta_{\mathrm{CL}}$, $\sigma^{2}$and $\omega_{\mathrm{CL}}^{2})$.

| **Scenario** | | **Model** | **CL [L/h]** | **Vd [L]** | **Residual error**  **(**$\boldsymbol{\sigma}^{\mathbf{2}}$**)** | **IIV on CL**  **(**$\boldsymbol{\omega}_{\mathbf{CL}}^{\mathbf{2}}$**)** |
| --- | --- | --- | --- | --- | --- | --- |
| i. | $\theta_{\mathrm{CL}}$ | M1-M6 | 1, 2, 4, 8, 16, 32 | 60 | 0.01 | 0.1 |
| ii. | $\sigma^{2}$ | M1-M6 | 3 | 60 | 0.01, 0.04, 0.09, 0.16, 0.25, 0.36 | 0.1 |
| iii. | $\omega_{\mathrm{CL}}^{2}$ | M1-M6 | 3 | 60 | 0.01 | 0.01, 0.04, 0.09, 0.16, 0.25, 0.36 |

**Supplementary Figure S1.** Visualization of simulated data across parameter misspecification scenarios ($\theta_{\mathrm{CL}}$, $\sigma^{2}$and $\omega_{\mathrm{CL}}^{2}$). Labels S1-S6 correspond to the sub-populations within each scenario generated from models M1-M6 with parameter values increasing incrementally from model M1-M6. In the $\theta_{\mathrm{CL}}$ misspecification scenario, clearance values range from 1- 32 L/h (1, 2, 4, 6, 8, 16, 32). In the $\sigma^{2}$ misspecification scenario, residual variability ($\sigma^{2}$) ranges from 0.01-0.36 (0.01, 0.04, 0.09, 0.16, 0.25, 0.36). In the $\omega^{2}$ misspecification scenario, interindividual variability on clearance ($\omega_{\mathrm{CL}}^{2}$) takes the same set of values as in $\sigma^{2}$. Approximated peak and trough observations (1-hour post start of infusion and 1 hour prior to following dose) following the 4^th^ dosing occasion (black) were used to inform, while those following the 6^th^ dosing occasion (grey) which were hidden from the model and used in Bayesian forecasting to asses predictive performance. A total of 6000 individuals (sub-populations S1-S6) were simulated per misspecification scenario.

## 1.2 Results

### 1.2.1 Goodness-of-fit plots

**Supplementary Figure S2.** Individual predicted concentrations (IPRED) vs. observed concentrations (DV) of the 6 individual models (M1-M6) and the re-estimated model (7) on a total population size of 6000, respective to their corresponding dataset within each model misspecification scenario ($\theta_{\mathrm{CL}}$, $\sigma^{2}$and $\omega_{\mathrm{CL}}^{2}$).

### 1.2.2 MAPE and MPE: raw results

**Supplementary Table S3.** Calculated mean absolute prediction error (MAPE%) and mean prediction error (MPE%) for the $\theta_{\mathrm{CL}}$ misspecification scenario (i.) in a one-compartment model evaluating predictive performance for single sub-models (M1-M6), a re-estimated model and the various combinations of sub-models integrated into the model averaging approach. Performance metrics (MAPE/MPE) calculated for each sub-population consisting of 1000 simulated IDs and for the full population (S1-S6) consisting of 6000 IDs.

|  | | **MAPE [%]** | | | | | | | **MPE [%]** | | | | | | |
| --- | --- | --- | --- | --- | --- | --- | --- | --- | --- | --- | --- | --- | --- | --- | --- |
|  | | **SUB-POPULATION** | | | | | | | | | | | | | |
| **MODEL** | | **S1** | **S2** | **S3** | **S4** | **S5** | **S6** | **S1-S6** | **S1** | **S2** | **S3** | **S4** | **S5** | **S6** | **S1-S6** |
| **Single candidate models** | | | | | | | | | | | | | | | |
| **M1** | | 9.20 | 10.72 | 14.06 | 41.85 | 79.14 | 113.21 | **25.74** | -1.10 | 6.68 | 11.52 | 41.85 | 79.14 | 113.21 | **24.82** |
| **M2** | | 11.67 | 9.50 | 9.27 | 10.10 | 15.02 | 57.66 | **12.94** | -9.18 | -2.15 | 1.94 | 5.11 | 13.00 | 57.66 | **4.95** |
| **M3** | | 14.73 | 10.60 | 9.46 | 9.36 | 10.94 | 19.98 | **11.59** | -13.88 | -6.80 | -1.74 | 1.61 | 5.85 | 19.01 | **-0.26** |
| **M4** | | 17.90 | 12.07 | 10.06 | 9.47 | 9.45 | 12.55 | **11.40** | -17.68 | -9.99 | -5.03 | -1.41 | 1.55 | 8.43 | **-4.00** |
| **M5** | | 20.79 | 14.08 | 10.86 | 10.01 | 9.62 | 9.68 | **11.75** | -20.59 | -12.60 | -7.98 | -4.24 | -1.47 | 1.74 | **-7.15** |
| **M6** | | 23.12 | 15.84 | 11.98 | 10.85 | 9.94 | 9.61 | **12.75** | -23.07 | -15.51 | -10.51 | -6.89 | -4.27 | -1.52 | **-9.74** |
| **Re-est.** | | 10.04 | 9.63 | 9.30 | 9.30 | 9.43 | 9.49 | **9.55** | -4.48 | -2.51 | -2.00 | -1.57 | -1.39 | -1.29 | **-2.21** |
| **Model Averaging** | | | | | | | | | | | | | | | |
| OFV | All | 9.88 | 9.19 | 9.29 | 9.37 | 9.45 | 9.55 | **9.47** | -3.80 | -0.93 | -1.59 | -1.48 | -1.55 | -0.94 | **-1.73** |
|  | M1-M2 | 9.88 | 9.10 | 9.28 | 10.07 | 15.04 | 58.44 | **12.49** | -3.72 | 0.17 | 2.08 | 5.11 | 13.18 | 58.44 | **6.27** |
|  | M1-M3 | 9.20 | 10.72 | 11.43 | 10.67 | 9.94 | 9.61 | **10.64** | -1.10 | 6.68 | 5.76 | -5.09 | -4.27 | -1.52 | **2.38** |
|  | M1, M6 | 9.89 | 9.17 | 9.35 | 9.35 | 10.94 | 20.00 | **10.22** | -3.80 | -0.88 | -0.86 | 1.62 | 5.86 | 19.02 | **0.09** |
|  | M3-M6 | 17.90 | 12.07 | 10.05 | 9.41 | 9.45 | 9.55 | **10.30** | -17.68 | -9.99 | -5.04 | -2.07 | -1.56 | -0.94 | **-4.18** |
|  | M4-M6 | 14.73 | 10.60 | 9.45 | 9.37 | 9.45 | 9.55 | **10.89** | -13.89 | -6.82 | -2.68 | -1.48 | -1.55 | -0.94 | **-5.82** |
|  | M5-M6 | 20.79 | 14.08 | 10.86 | 9.98 | 9.53 | 9.55 | **11.69** | -20.59 | -12.60 | -7.98 | -4.31 | -2.03 | -0.94 | **-7.63** |
|  | | | | | | | | | | | | | | | |
| SSE | All | 9.81 | 10.04 | 9.21 | 9.36 | 9.48 | 11.25 | **9.81** | -3.68 | -0.12 | -0.65 | -0.96 | 0.09 | 4.94 | **-0.31** |
|  | M1-M2 | 9.78 | 9.78 | 9.21 | 10.14 | 15.02 | 57.99 | **12.76** | -3.57 | 0.68 | 3.12 | 5.37 | 13.01 | 57.99 | **6.77** |
|  | M1-M3 | 9.20 | 10.49 | 10.19 | 10.53 | 9.94 | 9.61 | **10.76** | -1.17 | 5.62 | 0.95 | -5.62 | -4.24 | -1.52 | **3.46** |
|  | M1, M6 | 9.81 | 10.02 | 9.09 | 9.51 | 11.05 | 20.01 | **9.97** | -3.65 | 0.15 | 1.40 | 2.77 | 6.43 | 19.12 | **-1.09** |
|  | M3-M6 | 17.90 | 12.22 | 10.23 | 9.80 | 9.50 | 11.10 | **10.63** | -17.70 | -10.15 | -6.04 | -3.54 | -1.17 | 4.22 | **-3.60** |
|  | M4-M6 | 14.74 | 10.69 | 9.53 | 9.53 | 9.51 | 11.25 | **11.31** | -13.89 | -7.04 | -3.61 | -2.12 | -0.19 | 4.94 | **-5.62** |
|  | M5-M6 | 20.79 | 14.21 | 11.03 | 10.17 | 9.61 | 9.56 | **11.86** | -20.60 | -12.66 | -8.43 | -5.21 | -2.66 | 0.05 | **-7.88** |

**Supplementary Table S4.** Calculated mean absolute prediction error (MAPE%) and mean prediction error (MPE%) for the $\sigma^{2}$ misspecification scenario (ii.) in a one-compartment model evaluating predictive performance for single sub-models (M1-M6), a re-estimated model and the various combinations of sub-models integrated into the model averaging approach. Performance metrics (MAPE/MPE) calculated for each sub-population consisting of 1000 simulated IDs and for the full population (S1-S6) consisting of 6000 IDs.

|  | | **MAPE [%]** | | | | | | | **MPE [%]** | | | | | | |
| --- | --- | --- | --- | --- | --- | --- | --- | --- | --- | --- | --- | --- | --- | --- | --- |
|  | | **SUB-POPULATION** | | | | | | | | | | | | | |
| **MODEL** | | **S1** | **S2** | **S3** | **S4** | **S5** | **S6** | **S1-S6** | **S1** | **S2** | **S3** | **S4** | **S5** | **S6** | **S1-S6** |
| **Single candidate models** | | | | | | | | | | | | | | | |
| **M1** | | 9.29 | 17.96 | 26.26 | 34.36 | 42.79 | 50.17 | **25.14** | -1.98 | -2.20 | -1.79 | 0.07 | 2.33 | 8.13 | **-0.56** |
| **M2** | | 10.52 | 17.91 | 25.86 | 33.12 | 40.70 | 47.51 | **24.77** | -3.76 | -3.64 | -3.03 | -1.80 | 0.49 | 4.85 | **-2.49** |
| **M3** | | 11.99 | 18.70 | 25.62 | 32.93 | 39.91 | 45.71 | **25.40** | -5.56 | -5.13 | -4.51 | -2.71 | 0.51 | 4.07 | **-3.74** |
| **M4** | | 13.77 | 19.57 | 26.42 | 33.30 | 39.84 | 45.53 | **26.28** | -7.48 | -6.55 | -4.99 | -3.18 | -0.23 | 2.40 | **-4.94** |
| **M5** | | 15.53 | 20.46 | 27.28 | 33.53 | 39.61 | 45.70 | **27.20** | -8.81 | -7.75 | -6.58 | -3.80 | -1.83 | 0.48 | **-5.84** |
| **M6** | | 16.94 | 21.42 | 27.44 | 33.76 | 40.10 | 45.50 | **28.02** | -9.66 | -8.43 | -7.63 | -5.01 | -2.97 | -0.84 | **-6.97** |
| **Re-est.** | | 17.17 | 21.37 | 27.33 | 33.65 | 39.53 | 45.54 | **28.06** | -9.28 | -7.40 | -6.41 | -3.90 | -1.60 | 0.03 | **-5.96** |
| **Model Averaging** | | | | | | | | | | | | | | | |
| **OFV** | **All** | 11.40 | 18.70 | 25.97 | 32.87 | 39.70 | 45.66 | **25.08** | -4.92 | -4.41 | -4.05 | -2.85 | -0.96 | 1.44 | **-3.53** |
|  | **M1-M2** | 9.93 | 17.87 | 25.77 | 33.01 | 40.85 | 47.28 | **24.71** | -2.68 | -2.77 | -2.75 | -1.73 | 0.88 | 5.08 | **-1.76** |
|  | **M1-M3** | 10.80 | 18.49 | 26.39 | 32.87 | 39.35 | 45.82 | **24.38** | -3.26 | -3.68 | -4.07 | -3.10 | -1.81 | 0.78 | **-2.11** |
|  | **M1, M6** | 10.49 | 18.03 | 25.35 | 32.47 | 39.81 | 46.13 | **24.95** | -3.40 | -3.28 | -2.63 | -1.60 | 0.96 | 4.80 | **-3.07** |
|  | **M3-M6** | 15.22 | 20.13 | 27.17 | 33.31 | 39.79 | 45.62 | **26.39** | -8.57 | -7.69 | -6.33 | -3.57 | -1.91 | -0.06 | **-5.15** |
|  | **M4-M6** | 14.01 | 19.74 | 26.67 | 33.33 | 39.79 | 45.66 | **27.08** | -7.47 | -6.83 | -5.39 | -3.05 | -1.62 | 0.40 | **-5.79** |
|  | **M5-M6** | 16.12 | 20.84 | 27.30 | 33.86 | 40.00 | 45.23 | **27.60** | -9.21 | -8.05 | -7.03 | -4.46 | -2.06 | -0.56 | **-6.36** |
|  | | | | | | | | | | | | | | | |
| **SSE** | **All** | 9.47 | 17.92 | 26.05 | 34.11 | 42.18 | 49.21 | **24.85** | -1.98 | -2.08 | -1.52 | -0.68 | 1.44 | 3.98 | **-1.09** |
|  | **M1-M2** | 9.35 | 17.92 | 26.08 | 34.32 | 42.36 | 49.32 | **25.01** | -2.03 | -2.20 | -1.60 | -0.06 | 2.26 | 7.89 | **-0.70** |
|  | **M1-M3** | 9.31 | 17.95 | 26.14 | 34.19 | 42.26 | 49.36 | **24.95** | -1.97 | -2.20 | -1.51 | -0.68 | 1.43 | 4.35 | **-0.88** |
|  | **M1, M6** | 9.45 | 17.95 | 26.07 | 34.17 | 42.34 | 49.55 | **24.87** | -1.95 | -2.00 | -1.48 | -0.40 | 2.05 | 7.12 | **-0.97** |
|  | **M3-M6** | 13.93 | 19.66 | 26.51 | 33.58 | 39.95 | 45.83 | **25.35** | -7.63 | -6.58 | -5.17 | -3.32 | -1.62 | 0.28 | **-4.17** |
|  | **M4-M6** | 12.17 | 18.83 | 25.64 | 33.25 | 39.53 | 45.98 | **26.24** | -5.81 | -5.28 | -4.70 | -3.20 | -1.23 | 1.57 | **-5.19** |
|  | **M5-M6** | 15.59 | 20.41 | 27.32 | 33.72 | 39.71 | 45.76 | **27.27** | -8.87 | -7.76 | -6.70 | -3.92 | -2.11 | -0.22 | **-5.98** |

**Supplementary Table S5.** Calculated mean absolute prediction error (MAPE%) and mean prediction error (MPE%) for the $\omega_{\mathrm{CL}}^{2}$ misspecification scenario (iii.) in a one-compartment model evaluating predictive performance for single sub-models (M1-M6), a re-estimated model and the various combinations of sub-models integrated into the model averaging approach. Performance metrics (MAPE/MPE) calculated for each sub-population consisting of 1000 simulated IDs and for the full population (S1-S6) consisting of 6000 IDs.

|  | | **MAPE [%]** | | | | | | | **MPE [%]** | | | | | | |
| --- | --- | --- | --- | --- | --- | --- | --- | --- | --- | --- | --- | --- | --- | --- | --- |
|  | | **SUB-POPULATION** | | | | | | | | | | | | | |
| **MODEL** | | **S1** | **S2** | **S3** | **S4** | **S5** | **S6** | **S1-S6** | **S1** | **S2** | **S3** | **S4** | **S5** | **S6** | **S1-S6** |
| **Single candidate models** | | | | | | | | | | | | | | | |
| **M1** | | 8.97 | 9.90 | 10.93 | 12.09 | 13.42 | 14.98 | **11.32** | -0.71 | -0.58 | -0.63 | -0.49 | -0.47 | -0.51 | **-0.58** |
| **M2** | | 9.16 | 9.25 | 9.49 | 10.03 | 10.32 | 10.74 | **9.79** | -1.65 | -1.64 | -1.78 | -1.96 | -2.22 | -2.24 | **-1.94** |
| **M3** | | 9.18 | 9.17 | 9.27 | 9.52 | 9.86 | 9.97 | **9.48** | -1.88 | -1.85 | -1.95 | -1.97 | -2.03 | -2.35 | **-2.00** |
| **M4** | | 9.25 | 9.29 | 9.32 | 9.42 | 9.59 | 9.73 | **9.39** | -1.78 | -1.80 | -1.92 | -2.01 | -2.04 | -2.16 | **-1.94** |
| **M5** | | 9.31 | 9.34 | 9.34 | 9.46 | 9.52 | 9.64 | **9.45** | -1.62 | -1.62 | -1.69 | -1.75 | -1.96 | -2.19 | **-1.79** |
| **M6** | | 9.30 | 9.34 | 9.38 | 9.48 | 9.44 | 9.58 | **9.41** | -1.41 | -1.45 | -1.55 | -1.53 | -1.76 | -1.94 | **-1.65** |
| **Re-est.** | | 9.14 | 9.17 | 9.32 | 9.46 | 9.64 | 9.69 | **9.39** | -1.82 | -1.94 | -1.85 | -1.96 | -2.03 | -2.13 | **-1.96** |
| **Model Averaging** | | | | | | | | | | | | | | | |
| **OFV** | **All** | 9.15 | 9.20 | 9.38 | 9.47 | 9.57 | 9.62 | **9.43** | -1.34 | -1.63 | -1.71 | -1.98 | -2.13 | -2.38 | **-1.92** |
|  | **M1-M2** | 8.91 | 9.35 | 9.77 | 10.06 | 10.46 | 10.68 | **9.85** | -1.17 | -1.46 | -1.74 | -1.60 | -1.82 | -2.04 | **-1.61** |
|  | **M1-M3** | 8.93 | 9.32 | 9.53 | 9.63 | 9.71 | 9.74 | **9.61** | -0.94 | -1.27 | -1.42 | -1.54 | -1.68 | -1.92 | **-1.88** |
|  | **M1, M6** | 9.06 | 9.22 | 9.59 | 9.73 | 9.92 | 10.16 | **9.48** | -1.41 | -1.50 | -1.83 | -2.00 | -2.19 | -2.32 | **-1.46** |
|  | **M3-M6** | 9.26 | 9.31 | 9.31 | 9.40 | 9.52 | 9.63 | **9.37** | -1.64 | -1.66 | -1.73 | -1.87 | -1.95 | -2.09 | **-1.85** |
|  | **M4-M6** | 9.26 | 9.30 | 9.33 | 9.41 | 9.51 | 9.62 | **9.43** | -1.76 | -1.73 | -1.84 | -1.85 | -1.98 | -2.25 | **-1.83** |
|  | **M5-M6** | 9.29 | 9.33 | 9.34 | 9.45 | 9.56 | 9.66 | **9.42** | -1.50 | -1.51 | -1.70 | -1.72 | -1.92 | -2.01 | **-1.75** |
|  | | | | | | | | | | | | | | | |
| **SSE** | **All** | 9.15 | 9.17 | 9.36 | 9.40 | 9.49 | 9.59 | **9.38** | -1.43 | -1.48 | -1.47 | -1.56 | -1.65 | -1.79 | **-1.53** |
|  | **M1-M2** | 9.10 | 9.23 | 9.67 | 10.08 | 10.49 | 10.79 | **9.85** | -1.14 | -1.22 | -1.47 | -1.58 | -1.64 | -1.87 | **-1.49** |
|  | **M1-M3** | 9.16 | 9.21 | 9.31 | 9.38 | 9.42 | 9.55 | **9.52** | -1.10 | -1.22 | -1.36 | -1.42 | -1.64 | -1.84 | **-1.48** |
|  | **M1, M6** | 9.20 | 9.19 | 9.37 | 9.64 | 9.87 | 10.04 | **9.35** | -1.23 | -1.30 | -1.37 | -1.48 | -1.65 | -1.81 | **-1.49** |
|  | **M3-M6** | 9.28 | 9.32 | 9.39 | 9.41 | 9.49 | 9.60 | **9.41** | -1.58 | -1.57 | -1.64 | -1.70 | -1.85 | -1.99 | **-1.72** |
|  | **M4-M6** | 9.29 | 9.31 | 9.30 | 9.41 | 9.49 | 9.64 | **9.42** | -1.65 | -1.64 | -1.65 | -1.69 | -1.82 | -1.87 | **-1.71** |
|  | **M5-M6** | 9.29 | 9.33 | 9.36 | 9.41 | 9.53 | 9.60 | **9.41** | -1.46 | -1.50 | -1.69 | -1.67 | -1.84 | -1.97 | **-1.71** |

### 1.2.3 MAPE, MPE and PE: all possible combinations

**Supplementary Figure S3.** Model averaging combinations for the $\theta_{\mathrm{CL}}$ one-compartment misspecification scenario including all possible combinations including n=2 to n=4 using both the OFV and SSE weighting schemes.

### 1.2.4 rRMSE and rBias

Model performance was additionally assessed by calculating the relative root mean square error and the relative bias (rBias) as follows [1]:

$rBias=\frac{1}{n}\sum_{1}^{i} \left( \frac{C_{predicted,i}-C_{measured,i}}{C_{measured,i}} \right)*100\%$	(S1)

$rRMSE=\sqrt{\frac{1}{n}\sum_{1}^{i} \left( \frac{C_{predicted,i}-C_{measured,i}}{C_{measured,i}} \right)^{2}}*100\%$	(S2)

Where *n* represents the total number of patients and *i* is each individual patient.

**Supplementary Figure S4.** rRMSE% and rBias% values from the $\theta_{\mathrm{CL}}$, $\sigma^{2}$, and $\omega_{\mathrm{CL}}^{2}$ misspecification scenarios (i. - iii.) based on Bayesian predicted peak and trough concentrations following a 6^th^ dose. Results are shown for (1) single candidate models (models 1-6), (2) a re-estimated model (Re-est.) and (3) model averaging including various combinations of the candidate models, including all six models (M1-M6). Within each misspecification scenario, models 1-6 correspond to parameterizations in which the parameter of interest ($\theta_{\mathrm{CL}}$, $\sigma^{2}$, or $\omega_{\mathrm{CL}}^{2}$) was sequentially increased from lowest (M1) to highest (M6), while all other parameters were held constant. In the $\theta$ misspecification scenario, clearance values range from 1- 32 L/h (1, 2, 4, 6, 8, 16, 32). In the $\sigma^{2}$ misspecification scenario, residual variability ($\sigma^{2}$) ranges from 0.01-0.36 (0.01, 0.04, 0.09, 0.16, 0.25, 0.36). In the $\omega^{2}$ misspecification scenario, interindividual variability on clearance ($\omega_{\mathrm{CL}}^{2}$) takes the same set of values as in $\sigma^{2}$. Performance metrics (MAPE/MPE) were calculated on the full population level (n = 600; horizontal line) and on the level of each of the sub-populations (n= 100; shapes). Within model averaging, dark blue and light grey correspond to OFV and SSE weighting, respectively.

# 2. Extended simulation analysis: One- and Two-compartment Sensitivity Analysis

## 2.1 Overview

The following simulations are an extension of the one-compartment simulation study assessing the generalizability of the prior findings impact of the three model misspecification scenarios ($\theta$, $\sigma^{2}$and $\omega_{\mathrm{CL}}^{2}$) with a sensitivity analysis for the following:

(1) **Impact of sampling design:**
    a. One-compartment model structure
    b. Two-compartment model structure
(2) **Model structure cross-evaluation**: for single model performance in the case of structural misspecification

**Dataset simulation**

An additional dataset was simulated for both a one- and two compartment model structure per misspecification scenario according to the dosing template in Supplementary Table S6. The parameterization for each misspecification scenario is reported in Supplementary Table S7.

As in the primary simulation study, the $\theta_{\mathrm{CL}}$, $\sigma^{2}$and $\omega_{\mathrm{CL}}^{2}$ misspecification scenarios each comprised of 6 sub-models, with 1000 IDs per simulated sub-model, yielding a total population size of 6000 IDs per scenario per model structure (one- and two-compartment).

The structural misspecification scenario for the two-compartment model was extended from the prior $\theta_{\mathrm{CL}}$ misspecification scenario to additionally assess misspecified volume of the central compartment ($\theta_{\mathrm{Vd}}$) and misspecified intercompartmental clearance ($\theta_{Q}$), yielding a total of 10 sub-models and 10,000 simulated IDs.

**Forecasting Performance**

Predictive performance was evaluated using the Bayesian forecasting approach for the 4 sampling designs detailed in Supplementary Figures S5-S9). Performance was evaluated for: (1) single candidate models (models M1-M6), (2) a re-estimated model using first-order conditional estimation with interaction (FOCE-I) on the full (heterogenous) dataset containing simulated data from all six models (10 models for two-compartment $\theta$ misspecification scenario) and (3) various combinations of the six candidate models in MAA (conducted only for $\theta$ misspecification, showing the greatest impact in the primary simulation study).

(1) **Impact of sampling design:**

The simulated datasets, per compartmental model structure and misspecification scenario consisted of a densely sampled study where concentration samples were simulated in the 1^st^, 4^th^ and 6^th^ dosing intervals. The design enabled an evaluation of both dense and sparse sampling, using either the 1^st^ or 4^th^ dosing intervals to predict the concentrations in the 6^th^ dosing interval as shown in Supplementary Figures S5-S8. For the sparse sampling design using observations from the 1^st^ dosing interval, the “peak” was simulated at 0.5-hour post start of infusion, while in the 4^th^ and 6^th^ dosing intervals the “peak” was approximated at 1-hour post start of infusion, consistent with the original design. In the dense sampling design, all simulated samples in either the 1^st^ or 4^th^ dosing interval were used to predict all simulated concentration samples in the 6^th^ dosing interval as shown in Supplementary Figures S7-S8. Per sampling design, model predictive performance was assessed for both compartmental model structures:

a. one-compartmental structure:
   i. single model predictive performance for all three misspecification scenarios ($\theta_{\mathrm{CL}}$, $\sigma^{2}$and $\omega_{\mathrm{CL}}^{2}$)
   ii. model averaging predictive performance for $\theta_{\mathrm{CL}}$ misspecification scenario
b. two-compartmental structure:
   i. single model predictive performance for all three misspecification scenarios and extended structural misspecification scenario ($\theta$, $\sigma^{2}$and $\omega_{\mathrm{CL}}^{2}$)
   ii. model averaging predictive performance for $\theta$misspecification scenario ($\theta_{\mathrm{CL}}$, $\theta_{\mathrm{Vd}}$, $\theta_{Q}$)

(2) **Model structure cross-evaluation:**
    a. Assessment of one-compartment candidate models evaluated on the two-compartment generated dataset
    b. Assessment of two-compartment candidate models evaluated on the one-compartment generated dataset

## 2.2 Dataset simulation

**Supplementary Table S6.** Dosing template used to simulate datasets using one- and two-compartment model structures for all three misspecification scenarios ($\theta$, $\sigma^{2}$and $\omega_{\mathrm{CL}}^{2})$ to assess different sampling designs. Concentrations were simulated after the 1^st^, 4^th^ and 6^th^ dosing occasions. Following simulation of the datasets, the EVID and MDV were updated according to the sampling design demonstrated in Section 2.3 and observations in the 6^th^ dosing occasion were hidden from the model for Bayesian forecasting.

| **ID** | **TIME** | **CMT** | **EVID** | **AMT** | **RATE** | **DV** | **OCC** | **GROUP** | **MDV** |
| --- | --- | --- | --- | --- | --- | --- | --- | --- | --- |
| 1 | 0 | 1 | 1 | 2000 | 4000 | . | 1 | 1 | 1 |
| 1 | 0.5 | 1 | 0 | 0 | 0 | . | 1 | 1 | 0 |
| 1 | 1.0 | 1 | 0 | 0 | 0 | . | 1 | 1 | 0 |
| 1 | 2 | 1 | 0 | 0 | 0 | . | 1 | 1 | 0 |
| 1 | 5 | 1 | 0 | 0 | 0 | . | 1 | 1 | 0 |
| 1 | 8 | 1 | 0 | 0 | 0 | . | 1 | 1 | 0 |
| 1 | 10 | 1 | 0 | 0 | 0 | . | 1 | 1 | 0 |
| 1 | 11 | 1 | 0 | 0 | 0 | . | 1 | 1 | 0 |
| 1 | 12 | 1 | 1 | 1000 | 2000 | . | 2 | 1 | 1 |
| 1 | 24 | 1 | 1 | 1000 | 2000 | . | 3 | 1 | 1 |
| 1 | 36 | 1 | 1 | 1000 | 2000 | . | 4 | 1 | 1 |
| 1 | 37 | 1 | 0 | 0 | 0 | . | 4 | 1 | 0 |
| 1 | 38 | 1 | 0 | 0 | 0 | . | 4 | 1 | 0 |
| 1 | 41 | 1 | 0 | 0 | 0 | . | 4 | 1 | 0 |
| 1 | 44 | 1 | 0 | 0 | 0 | . | 4 | 1 | 0 |
| 1 | 41 | 1 | 0 | 0 | 0 | . | 4 | 1 | 0 |
| 1 | 46 | 1 | 0 | 0 | 0 | . | 4 | 1 | 0 |
| 1 | 47 | 1 | 0 | 0 | 0 | . | 4 | 1 | 0 |
| 1 | 48 | 1 | 1 | 1000 | 2000 | . | 5 | 1 | 1 |
| 1 | 60 | 1 | 1 | 1000 | 2000 | . | 6 | 1 | 1 |
| 1 | 61 | 1 | 0 | 0 | 0 | . | 6 | 1 | 0 |
| 1 | 71 | 1 | 0 | 0 | 0 | . | 6 | 1 | 0 |

## 2.3 Sampling Design

 **Supplementary Figure S5.** Schematic describing the sparse sampling design using observations from the 1^st^ dosing interval (OCC=1) for Bayesian forecasting to predict concentrations in the 6^th^ dosing interval (OCC=6).

**Supplementary Figure S6.** Schematic describing the sparse sampling design using observations from the 4^th^ dosing interval (OCC=1) for Bayesian forecasting to predict concentrations in the 6^th^ dosing interval (OCC=6).

**Supplementary Figure S7.** Schematic describing the dense sampling design using observations from the 1^st^ dosing interval (OCC=1) for Bayesian forecasting to predict concentrations in the 6^th^ dosing interval (OCC=6).

**Supplementary Figure S8.** Schematic describing the dense sampling design using observations from the 4^th^ dosing interval (OCC=1) for Bayesian forecasting to predict concentrations in the 6^th^ dosing interval (OCC=6).

## 2.4 **One- and two compartment model** parameterizations

**Supplementary Table S7.** Model parameterization of one- and two-compartment models for sampling evaluation and cross-evaluation of structural model comparison scenarios.

| **Scenario** | | **Comp.** | **Model** | **CL [L/h]** | **Vd [L]** | **Q [L/h]** | **Vp [L]** | **Residual error (**$\boldsymbol{\sigma}^{\mathbf{2}}$**)** | **IIV on CL (**$\boldsymbol{\omega}_{\mathbf{CL}}^{\mathbf{2}}$**)** |
| --- | --- | --- | --- | --- | --- | --- | --- | --- | --- |
| i. | $\theta_{\mathrm{CL}}$ | 1-cmt | M1-M6 | 1, 2, 4, 8, 16, 32 | 60 | - | - | 0.01 | 0.1 |
|  | $\theta_{\mathrm{CL}}$ | 2-cmt | M1-M6 | 1, 2, 4, 8, 16, 32 | 60 | - | - | 0.01 | 0.1 |
|  | $\theta_{\mathrm{Vd}}$ | 2-cmt | M7-M8 | 3 | 30, 90 | 10 | 25 | 0.01 | 0.1 |
|  | $\theta_{Q}$ | 2-cmt | M9-M10 | 3 | 60 | 2, 20 | 25 | 0.01 | 0.1 |
| ii. | $\sigma^{2}$ | 1- and 2-cmt | M1-M6 | 3 | 60 | 10 | 25 | 0.01, 0.04, 0.09, 0.16, 0.25, 0.36 | 0.1 |
| iii. | $\omega_{\mathrm{CL}}^{2}$ | 1- and 2-cmt | M1-M6 | 3 | 60 | 10 | 25 | 0.01 | 0.01, 0.04, 0.09, 0.16, 0.25, 0.36 |

## 2.5 **Results**

## 2.5.1 Impact of sampling design

## 2.5.1.1 One-compartment sampling evaluation: single model predictive performance

**Supplementary Figure S9.** MAPE% and MPE% values for single model predictions (M1-M6 and a re-estimated model, re-est.) from the one-compartment $\theta_{\mathrm{CL}}$, $\sigma^{2}$, and $\omega_{\mathrm{CL}}^{2}$ misspecification scenarios (i. - iii.) evaluating the predictive performance in Bayesian forecasting to predict observations in the 6^th^ dosing interval (OCC = 6) under each of the four sampling designs: (1) dense sampling using 1^st^ dose observation (OCC = 1), (2) dense sampling using 4^th^ dose observation (OCC = 4), (3) sparse sampling using 1^st^ dose observation (OCC = 1), and (4) sparse sampling using 4^th^ dose observation (OCC = 4).

### 2.5.1.2 One-compartment sampling evaluation: model averaging

**Supplementary Figure S10.** MAPE% and MPE% values for model averaging predictions (OFV and SSE weighting) for the full population from the one-compartment $\theta_{\mathrm{CL}}$ misspecification scenarios (i.) evaluating the predictive performance in Bayesian forecasting to predict observations in the 6^th^ dosing interval (OCC = 6) under each of the four sampling designs: (1) dense sampling using 1^st^ dose observation (OCC = 1), (2) dense sampling using 4^th^ dose observation (OCC = 4), (3) sparse sampling using 1^st^ dose observation (OCC = 1), and (4) sparse sampling using 4^th^ dose observation (OCC = 4).

### 2.5.1.3 Two-compartment sampling evaluation: single model predictive performance

**Supplementary Figure S11.** MAPE% and MPE% values for single model predictions (M1-M10 and a re-estimated model, re-est.) for the full population from the two-compartment $\theta$misspecification scenarios ($\theta_{\mathrm{CL}}, \theta_{\mathrm{Vd}}$, and $\theta_{Q}$). Models 1-6 (M1-M6) correspond to subsequently increasing CL values for $\theta_{\mathrm{CL}}$ misspecification (1 – 32 L/h), models 7 and 8 (M7 and M8) correspond to$\theta_{\mathrm{Vd}}$ misspecifications (30 and 90 L, respectively) and models 9 and 10 (M1 and M10) correspond to $\theta_{Q}$ misspecification (2 and 20 L/h, respectively). Each single model was assessed by evaluating its predictive performance in Bayesian forecasting of observations in the 6^th^ dosing interval (OCC = 6) under each of the four sampling designs: (1) dense sampling using 1^st^ dose observation (OCC = 1), (2) dense sampling using 4^th^ dose observation (OCC = 4), (3) sparse sampling using 1^st^ dose observation (OCC = 1), and (4) sparse sampling using 4^th^ dose observation (OCC = 4).

**Supplementary Figure S12.** MAPE% and MPE% values for single model predictions (M1-M6 and a re-estimated model, re-est.) for the full population from the two-compartment $\sigma^{2}$ and $\omega_{\mathrm{CL}}^{2}$ misspecification scenarios. Models 1-6 (M1-M6) increasing residual variability ($\sigma^{2}$) and interindividual variability on clearance ($\omega_{\mathrm{CL}}^{2}$) with ranges from 0.01-0.36 (0.01, 0.04, 0.09, 0.16, 0.25, 0.36). Each single model was assessed by evaluating its predictive performance in Bayesian forecasting of observations in the 6^th^ dosing interval (OCC = 6) under each of the four sampling designs: (1) dense sampling using 1^st^ dose observation (OCC = 1), (2) dense sampling using 4^th^ dose observation (OCC = 4), (3) sparse sampling using 1^st^ dose observation (OCC = 1), and (4) sparse sampling using 4^th^ dose observation (OCC = 4).

### 2.5.1.4 Two-compartment sampling evaluation: model averaging

**Supplementary Figure S13.** MAPE% and MPE% values for model averaging predictions (OFV and SSE weighting) for the full population from the two-compartment $\theta$misspecification scenarios ($\theta_{\mathrm{CL}}$, $\theta_{\mathrm{Vd}}$, and $\theta_{Q}$) evaluating the predictive performance in Bayesian forecasting to predict observations in the 6^th^ dosing interval (OCC = 6) under each of the four sampling designs: (1) dense sampling using 1^st^ dose observation (OCC = 1), (2) dense sampling using 4^th^ dose observation (OCC = 4), (3) sparse sampling using 1^st^ dose observation (OCC = 1), and (4) sparse sampling using 4^th^ dose observation (OCC = 4).

### 2.5.2 Model structure cross evaluation: $\boldsymbol{\theta}$ misspecification

**Supplementary Figure S14.** Structural model cross-evaluation using observations from the 4^th^ occasion under the sparse sampling design. The “original design” corresponds to the one-compartment candidate models evaluated on the dataset generated under the one-compartment structure, while the “cross-evaluation is the one-compartment candidate models evaluated on the two-compartment generated dataset.

**Supplementary Figure S15.** Structural model cross-evaluation using the 4^th^ occasion observations under the sparse sampling design. The “original design” corresponds to the two-compartment candidate models evaluated on the dataset generated under the two-compartment structure, while the “cross-evaluation is the two-compartment candidate models evaluated on the one-compartment generated dataset.

**Supplementary Code S1.**

**NM-TRAN Control Stream**

The following code shows the one-compartmental model structure used for all 3 scenarios. The parameterization of models 1-6 for each misspecification scenario is according to Table S2. For model re-estimation, MAXEVAL in the following code was change to a value of 9999.

$PROBLEM

$DATA theta_NM.csv	IGNORE=@

$INPUT ID TIME CMT EVID AMT RATE DV OCC GROUP MDV

$SUBROUTINES ADVAN1 TRANS2

$PK

TVCL = THETA(1)

TVV	= THETA(2)

CL = TVCL * EXP(ETA(1)) ; clearance [L/hr]

V	= TVV * EXP(ETA(2)) ; volume of distribution [L]

S1 = V

$ERROR

IPRED = F	; individual-specific prediction

Y = IPRED + IPRED * EPS(1)

W = SQRT(SIGMA(1) * IPRED * IPRED) ; for proportional error model

IRES = DV - IPRED

IWRES = IRES/(W+0.0001)

$THETA

1.0	; 1_CL

60.0	; 2_V1

$OMEGA

0.1	; 1_IIV_CL

0.1	; 2_IIV_V1

$SIGMA

0.01	; 1_PropErr

$ESTIMATION METHOD=1 INTERACTION MAXEVAL=0 SIG=3 PRINT=1 NOABORT

$TABLE ID TIME DV EVID IPRED CL V ETAS(1:LAST) IWRES CWRES NPDE ONEHEADER NOPRINT FILE=sdtab001

$TABLE ID CL V ETAS(1:LAST) ONEHEADER NOPRINT FILE=patab001

**Supplementary Code S2.**

**NM-TRAN Control Stream**

The following code shows the two-compartmental model structure used for all 3 scenarios. The parameterization of models 1-10 for the $\theta$ misspecification scenario and models 1-6 for the $\sigma^{2}$and $\omega_{\mathrm{CL}}^{2}$ misspecification scenarios is according to Table S7. For model re-estimation, MAXEVAL in the following code was change to a value of 9999.

$PROBLEM

$DATA theta_NM.csv	IGNORE=@

$INPUT ID TIME CMT EVID AMT RATE DV OCC GROUP MDV

$SUBROUTINES ADVAN3 TRANS4

$PK

TVCL = THETA(1)

TVV1 = THETA(2)

TVQ	= THETA(3)

TVV2 = THETA(4)

CL = TVCL*EXP(ETA(1)) ; clearance [L/hr]

V1 = TVV1*EXP(ETA(2)) ; volume of distribution [L]

Q = TVQ	; inter-compartmental clearance [L/h]

V2 = TVV2	; volume of peripheral compartment [L]

S1 = V1

$ERROR

IPRED = F	; individual-specific prediction

Y = IPRED + IPRED * EPS(1)

W = SQRT(SIGMA(1) * IPRED * IPRED) ; for proportional error model

IRES = DV - IPRED

IWRES = IRES/(W+0.0001)

$THETA

3.0	;1_CL

60.0	;2_V1

10.0	;3_Q

25.0	;4_V2

$OMEGA

0.1	; 1_IIV_CL

0.1	; 2_IIV_V1

$SIGMA

0.01	; 1_PropErr

$ESTIMATION METHOD=1 INTERACTION MAXEVAL=0 SIG=3 PRINT=1 NOABORT

$TABLE ID TIME DV EVID IPRED CL V1 Q V2 ETAS(1:LAST) IWRES CWRES NPDE ONEHEADER NOPRINT FILE=sdtab001
